# Valuating the efficiency of social security and healthcare in OECD countries from a sustainable development

**DOI:** 10.1186/s12962-024-00555-y

**Published:** 2024-06-26

**Authors:** Li-wen Lee, Yung-ho Chiu, Fan-peng Liu, Tai-Yu Lin, Tzu-Han Chang

**Affiliations:** 1 Department of Economics, Soochow University, 56, Kueiyang St., Sec. 1, Taipei, 10048 Taiwan, ROC; 2 Department of Business Administration, National Cheng Kung University, No. 1, University Road, Tainan City, 701 Taiwan, ROC

**Keywords:** Social security efficiency, Healthcare efficiency, Sustainable development, Data envelopment analysis (DEA), Undesirable output

## Abstract

Under the goal of sustainable development, coping with the increase in social security and healthcare expenses caused by population aging is becoming increasingly important, but it is rare in the literature to evaluate the impact of social security efficiency on healthcare efficiency. This research uses the dynamic SBM two-stage model to observe the efficiencies of social security and healthcare in OECD countries. There are two findings as follows. First, the higher social security efficiency is, the better is the healthcare efficiency of countries with lower per capita GDP. Second, higher social security efficiency of National Health Service (NHS) countries denote better healthcare efficiency. When the financial source of the social security system is taxation, then it is more likely to bring higher efficiency to healthcare.

## Introduction

This research assesses the efficiency of social security and healthcare in OECD countries from a sustainable development perspective, enabling the transformation of resources into more successful operations. According to OECD Health Statistics 2022 [44], healthcare expenditures as a share of GDP across the organization’s countries have jumped from 7.8% in 2005 to 9.9% in 2020. Global healthcare challenges, as highlighted by Yaya and Danhoundo [63], include aging populations, cost control, widening health inequalities, and the shift from acute to chronic diseases, resulting in increased healthcare burdens and fragmented healthcare systems. These factors have prompted significant changes in healthcare systems in OECD countries over the past decade. Samut and Cafri [51] noted that almost all countries around the world face budget cuts in health spending, which force public and private hospitals in these countries to use their resources more effectively and to provide more efficient healthcare. Additionally, the share of social expenditures in terms of GDP increased from 20.1% in 2005 to 23.0% in 2020. Adams et al. [1] highlighted that social security and welfare are the largest public expenditure items, and the public sector’s effective operation is a necessary condition for a country’s economic performance. As both social security and healthcare expenditures are linked to aging populations, which is an ongoing trend, the United Nations introduced the Sustainable Development Goals (SDGs) in 2015, encompassing economic, social, and environmental aspects. SDG1 aims for no poverty, SDG3 focuses on good health and well-being, and SDG10 targets to reduce inequality, all of which relate to social security and healthcare. In this context, we evaluate healthcare and social security efficiency from a sustainable development perspective and explore whether the efficiency of social security operations affects healthcare efficiency.

A data envelopment analysis (DEA) literature review has found that social security efficiency is often approached from the perspective of the public sector or public health [4, 42]. On the other hand, the healthcare efficiency literature includes extensive cross-national comparisons [19, 24, 51]. The assessment of social security has often focused on individual countries or regions, as seen in Meena and Shiv [39]. In healthcare efficiency studies, it is common to include both healthcare and non-healthcare variables in a single model, as observed in Greene [19] and Ortega et al. [45]. Given that social security encompasses healthcare and differs fundamentally from healthcare provision, for a clear evaluation, our model distinguishes overall efficiency into two stages: social security and healthcare. This allows us to observe the impact of social security on healthcare. Mackenbach [37] found that European countries with high per capita healthcare expenditure did not show a high level of mortality reduction in the early 1980s, and there may be great differences in the cost-effectiveness of healthcare systems. Ozcan and Khushalani [46] noted that OECD countries with efficient public healthcare systems generally have better overall healthcare system efficiency.

From the Organization for Economic Cooperation and Development (OECD) as National Health Service (NHS) and National Health Insurance (NHI) [43], the appendix details the classification of health systems of each OECD country. Using the median per capita GDP (PPP-based) of the sample countries at US$41,500 sourced from the OECD database, we divide the countries into two groups: those with per capita GDP above US$41,500 and those with it below US$41,500. Employing the data, the study observes the efficiency values of social security and healthcare, as well as the higher and lower groups between NHS and NHI. We also include sustainable development indicator variables in the model to evaluate the overall efficiency, social security efficiency, and healthcare efficiency.

The contributions of this study are as follows. (1) Taking a first-differentiation of overall efficiency into social security and healthcare stages for a separate evaluation of the two stages’ efficiency. (2) Evaluating the efficiencies of social security and healthcare based on sustainable development indicators, and more specifically, no poverty (SDG1), good health and well-being (SDG3), and reduce inequality (SDG10). (3) Examining social security and healthcare efficiencies of countries with different healthcare systems and different levels of GDP, in relation to preventable and treatable mortality (SDG3.4) and maternal mortality (SDG3.1). The choice of OECD countries as evaluation objects is based on their status as major economic entities, and there is not much heterogeneity in terms of economic and social aspects, making cross-national comparisons meaningful.

We use data from 2012 to 2016 in 26 OECD countries and propose a two-stage recycle dynamic undesirable SBM DEA model. In stage 1, we examine the social security efficiency of each country, focusing on the social security stage. The input variables are number of insured by government social health insurance and net total social security expenditure, and the output variables are net social security benefits and SDG3.4 preventable and treatable mortality. The social protection expenditure per inhabitant links stage 1 to stage 2. In stage 2, we investigate the healthcare efficiency. The input variables are physicians and healthcare expenditure, and the output variables are hospital discharges, deaths (undesirable output), and SDG3.1 maternal mortality (undesirable output). Live births form the output of the healthcare stage that circulate back as inputs to the social security stage. The population serves as a dynamic carry-over variable. This study analyzes and discusses the following specific directions.

1. Compare social security and healthcare efficiency among different countries.
2. Explore the correlation between social security stage efficiency and healthcare stage efficiency.
3. Provide suggestions on the constituent factors of healthcare ineffectiveness and how to improve healthcare efficiency.
4. Analyze the optimal efficiency levels of SDG3.4 preventable and treatable mortality.

Many studies in the DEA literature have explored the efficiency of social security or healthcare. In terms of social security, there are Lavado et al. [33] and Nistor et al. [42] for single countries and Hayes et al. [22], Geys [17], Borge et al. [7], Adams et al. [1], and Antonelli and Bonis [4] for transnational parts. Regarding healthcare, single country studies include Nicola et al. [41] and Gearhart [16], while transnational parts studies cover Bhat [5], Samut and Cafri [51], Ozcan and Khushalani [46], and Top et al. [60]. In terms of stage classification, most healthcare efficiency assessments utilize a single-stage approach, as seen in studies by Steinmann et al. [54], Bhat [5], and Spinks and Hollingsworth [53]. Adams et al. [1] verified that government is the most significant factor influencing public sector efficiency.

The traditional DEA aims to represent efficiency as achieving higher outputs with fewer inputs. However, in reality there are undesirable outputs to consider. The healthcare efficiency literature considering these undesirable outputs encompasses Grosskopf et al. [20] and Hu et al. [25]. Hu et al. [25] argued that without considering undesirable output, the average efficiency score is overestimated and the efficiency ranking across provinces changes considerably. Relative to the variable for preventable and avoidable mortality, Rutstein et al. [50] examined the impact of socioeconomic status on healthcare outcomes. Lagasse et al. [32] and Poikolainen and Eskola [47] found higher avoidable mortality rates in socioeconomically deprived areas. Lagasse et al. [32] demonstrated the inequities in healthcare based on socioeconomic status through avoidable mortality. Treuniet et al. [61] noted that regions with more healthcare resources have lower mortality from preventable diseases, while Mackenbach et al. [37] partially supported these findings. Poikolainen and Eskola [47] did not find a significant correlation between the two factors.

Reviewing the SBM literature, Cooper et al. [12] used a non-radial efficiency measurement approach based on slack variables. Herrero et al. [23] assessed the mixed efficiency of multi-species fishing vessel operations. Liu and Wang [34] employed DEA to measure the Malmquist productivity of semiconductor packaging and testing companies in Taiwan from 2000 to 2003, obtaining more accurate results using SBM and Super-SBM. Chiu and Chen [10] evaluated bank efficiency using a three-stage method, with Super-SBM employed in the first stage to assess efficiency values considering internal risks. Drake et al. [14] used SBM to assess the efficiency of Japanese banks. Kritikos et al. [31] employed CRS, VRS, and SBM approaches to evaluate the relative efficiency of real estate firms across decision-making units (DMUs). Chiu et al. [11] utilized BCC model and Super-SBM to investigate whether there are significant differences in bank technical efficiency. So far, the use of SBM in social security and healthcare efficiency has been relatively limited. In the literature on social security efficiency, Meena and Shiv [39] examined social security efficiency in India using SBM and Super-SBM. Liu et al. [35] evaluated the efficiency of rural healthcare expenditure in China using Super-SBM and MPI approaches. In the healthcare sector, Zhang et al. [64] analyzed the efficiency of public hospitals in Japan. Shoaib [52] investigated the operational efficiency of health insurance companies in India. Hsu [24] employed SBM and Super-SBM to assess the performance of healthcare expenditure in Europe and Central Asia. Lu et al. [36] evaluated energy, healthcare efficiency, and productivity changes from 2011 to 2015.

The literature on social security efficiency primarily has focused on the public sector or public health perspectives typically examining a single country or region and thus making it difficult to observe differences across countries. While the research direction is linked to sustainable development, there is limited incorporation of sustainable development sub-goals as variables in the models. Reviewing the variables for preventable and avoidable mortality rates, we note that there is no consensus on whether different socioeconomic factors contribute to healthcare inequality across regions. Additionally, it is recognized that countries with highly efficient public healthcare systems generally exhibit better healthcare efficiency. Furthermore, well-being can alleviate the burden of healthcare costs and improve healthcare efficiency, highlighting the important role of governments in enhancing healthcare efficiency. In the public health efficiency literature, many studies incorporated social security and healthcare variables into a single model. However, it is unclear whether such an approach adequately considers the functional differences.

Most healthcare efficiency literature has overall focused on healthcare expenditure and efficiency discussions, with relatively less attention given to the impact of social security investment on healthcare efficiency. There is scant literature that has employed Two-stage Recycle Dynamic Undesirable SBM DEA to evaluate social security and healthcare efficiency, and no investigation has been conducted on whether the operational efficiency of social security affects healthcare efficiency. We use deaths and maternal mortality as undesirable outputs in the social security stage. Maternal mortality also aligns with SDG 3.1. Additionally, for the first time, countries are classified based on different levels of per capita GDP and different healthcare systems to conduct the analysis herein.

## Research methods

Tone [56] proposed the SBM (Slacks-Based Measures) model, which uses the slack variable as the measurement basis and considers the slack between input and output terms. SBM efficiency is presented using a non-radial estimation method and a scalar representation. Chen and Zhu [8], Kao and Hwang [29], and Kao [30] introduced a two-stage DEA model that divides the entire operational process into subprocesses and connects them through intermediate outputs. They calculated the efficiency of each stage under different conditions. Tone and Tsutsui [57] further developed the weighted SBM network DEA model, which utilizes the linkage between departments within DMUs as the analytical basis for the network DEA model and uses SBM to obtain the optimal solution. In the network DEA model, dynamic methods are allowed, where DMUs are evaluated at different time periods, and carry-over activities are introduced to connect the different stages of DMUs Tone and Tsutsui [58] for evaluating efficiency models across multiple periods. Tone and Tsutsui [59] proposed the weighted SBM Dynamic Network DEA model with the linkage among various departments of DMUs taken as the analysis basis of the Network DEA model and each department is regarded as a Sub-DMU. Carry-over activities are taken as the linkage.

Research on the circular economy is constantly evolving, particularly in recent years, as researchers and practitioners seek to understand how to measure and quantify its impacts in real-world settings. Scholars have used DEA to estimate circular economy efficiency in different regions or industries to demonstrate its performance [62]. However, the aforementioned DEA methods fail to describe the internal structure of circular economy systems, leading to biased evaluation results. To overcome this limitation, Sun et al. [55] first constructed a network game DEA method and attempted to model the closed-loop network structure of the circular economy. They improved the efficiency assessment of the circular economy. However, despite the focus of the literature on static analysis of circular economy performance, there is still a lack of comprehensive understanding of the dynamic changes in circular economy efficiency over time. Exploring efficiency growth modes, evolutionary trends, and internal dominance positions within the circular economy framework is of greater importance. Due to the lack of consideration for circular economy factors in the dynamic network DEA model proposed by Tone and Tsutsui [59], this study introduces two-stage recycle dynamic undesirable SBM DEA.

### Two-stage recycle dynamic undesirable SBM DEA

Suppose there are n OECD countries as DMUs (decision-making units) (o $=$ 1,…,n), k stages (k $=$ 1,…,K), and T time periods (t $=$ 1,…,T). Each DMU (OECD country) has its own set of inputs and outputs for each time period t and is connected to the next period t + 1 through a carry-over factor. Let ${m}_{k}$ and ${r}_{k}$ represent the inputs and outputs for each stage k, respectively, with ${(k,h)}_{i}$ denoting the stages from k to h, and ${L}_{hk}$ serving as the division set between k and h. The definitions of inputs, outputs, links, and carry-over are outlined as follows. Table 1 shows mathematical symbol.

**Table 1:** Mathematical symbol table

Symbol	Variable
${X}_{io1}^{t}$	Insured by government social health insurance
	Net social protection expenditure
${y}_{ro1}^{t}$	Net social protection benefits
	Treatable and preventable mortality
${Z}_{do{\left(\mathrm{1,2}\right)}}^{t}$	Social protection expenditure per inhabitant
${X}_{io2}^{t}$	Physicians
	Healthcare expenditure
${y}_{ro2good}^{t}$	Hospital discharges
${y}_{ro2bad}^{t}$	Maternal mortality
	Deaths
${Z}_{do{\left(\mathrm{2,1}\right)}}^{t}$	Live births
${Z}_{co{k}_{l}}^{\left(t,t+1\right)}$	Population
${W}^{t}$	Weight to period t
${W}^{k}$	Weight to division k

### Inputs and outputs

${X}_{iok}^{t}\upepsilon {R}_{+} (i=1,\dots ,{m}_{k}; o=1,\dots ,n; k=1,\dots ,K; t=1,\dots ,T)$ refers to input $i$ at time period $t$ for ${DMU}_{j}$ division $k$. ${X}_{iok}^{t}$ In stage 1 (social security), the input variables used are those insured under government social health insurance and net social protection expenditure. In stage 2 (healthcare), the input variables used are physicians and healthcare expenditure.

${Y}_{rok}^{t}\upepsilon {R}_{+} (r=1,\dots ,{r}_{k}; o=1,\dots ,n; k=1,\dots ,K; t=1,\dots ,T)$ refers to output r in time period $t$ for ${DMU}_{j}$ division $k$. In stage 1 (social security), the output variables used are net social security benefits and preventable and treatable mortality. In stage 2 (healthcare), deaths and maternal deaths are considered undesirable outputs, while hospital discharges is considered a desirable output.

### Links

${Z}_{do{\left(kh\right)}}^{t}\upepsilon {R}_{+}(d=1\dots .D,o=1,\dots ,n;(kh)=1,\dots , (KH); t=1,\dots ,T$) 0 refers to the period t links from ${DMU}_{j}$ division $k$ to division $h$, with ${\left(kh\right)}$ being the number of $k$ to $h$ links.

Stage 1 (social security) link stage 2 (healthcare); ${Z}_{d0{\left(\mathrm{1,2}\right)}}^{t}$: per capita social protection expenditure is selected as the link indicator in stage 1 and stage 2.

Stage 2 (healthcare) link stage 1(social security); ${Z}_{d0{\left(\mathrm{2,1}\right)}}^{t}$: live births is selected as the link indicator in stage 2 and stage 1.

### Carry-over

${Z}_{co{k}_{l}}^{\left(t,t+1\right)}\upepsilon {R}_{+}({\text{c}}=1\dots .{\text{C}}; o=1,\dots ,n;{k}_{l}=1,\dots , {K}_{l}; t=1,\dots ,T-1)$ refers to the carry-over of $t$ to the $t+1$ period from ${DMU}_{0}$ division $k$ to division$h$, with ${L}_{k}$ being the number of carry-over items in division $k$. The carry-over factor is population.

### Other variables

${W}^{t}(t=1\dots T)$ Is the weight to period t, and ${W}^{k}(k=1\dots k)$ is the weight to division k.

As each DMU under the chooses the most favorable final weighted output, the DMU efficiencies are solved using the following equations:

$${\theta }_{0}^{*}={\text{min}}\frac{{\sum }_{t=1}^{T}{W}^{t}\left[{\sum }_{k=1}^{K}{W}^{k}\left[1-\frac{1}{{m}_{k}+{ninput}_{k}}({\sum }_{i=1}^{{m}_{k}}\frac{{S}_{iok}^{t-}}{{x}_{iok}^{t}}+{\sum }_{{k}_{l}}^{{ninput}_{k}}\frac{{s}_{o{k}_{l}}^{(t,t+1)}}{{z}_{o{k}_{l}}^{(t,t+1)}})\right]\right]}{{\sum }_{t=1}^{T}{W}^{t}\left[{\sum }_{k=1}^{K}{W}^{k}\left[1+\frac{1}{{r}_{1k}+{r}_{2k}+{link}_{k}}(\sum_{r=1}^{{r}_{1k}}\frac{{s}_{rokgood}^{t+}}{{y}_{rokgood}^{t}}+\sum_{r=1}^{{r}_{2k}}\frac{{s}_{rokbad}^{t-}}{{y}_{rokbad}^{t}}+ {\sum }_{(kl)}^{link}\frac{{s}_{o(kl)}^{t}}{{Z}_{o(kl)}^{t}})\right]\right]}$$

with $${\sum }_{t=1}^{T}{W}^{t}=$$ 1; $${\sum }_{k=1}^{K}{W}^{k}=$$ 1.

Subject to:

Stage 1: social security stage

$${X}_{io1}^{t}=\sum_{o=1}^{n}{X}_{io1}^{t}{\lambda }_{io1}^{t}+{s}_{io1}^{t-}(i=1,\dots ,{m}_{k},)$$

$${y}_{ro1}^{t}=\sum_{o=1}^{n}{y}_{ro1}^{t}{\lambda }_{ro1}^{t}-{s}_{ro1}^{t+}(r=1,\dots ,{r}_{k})$$

$${\uplambda }_{i{\text{o}}1}^{{\text{t}}}\ge 0, { {\uplambda }_{r{\text{o}}1}^{{\text{t}}}\ge 0;s}_{io1}^{t-}\ge 0, { s}_{ro1}^{t+}\ge 0$$

$${Z}_{do{\left({1,2}\right)}}^{t}=\sum_{o=1}^{n}{Z}_{do{\left(1, 2\right)}}^{t}{\uplambda }_{{\text{do}}(1, 2)}^{{\text{t}}}-{s}_{do{\left(1, 2\right)}}^{t-}$$(d $$=$$ 1…D)

Stage 2: healthcare stage

$${X}_{io2}^{t}=\sum_{o=1}^{n}{X}_{io2}^{t}{\lambda }_{io2}^{t}+{s}_{io2}^{t-}(i=1,\dots ,{m}_{k})$$

$${y}_{ro2good}^{t}=\sum_{o=1}^{n}{y}_{ro2good}^{t}{\lambda }_{ro2good}^{t}-{s}_{ro2good}^{t+}(r=1,\dots ,{r}_{k})$$

$${y}_{ro2bad}^{t}=\sum_{o=1}^{n}{y}_{ro2bad}^{t}{\lambda }_{ro2bad}^{t}+{s}_{ro2bad}^{t+}(r=1,\dots ,{r}_{k})$$

$${\uplambda }_{i{\text{o}}2.1}^{{\text{t}}}\ge 0, { {\uplambda }_{r{\text{o}}2.1}^{{\text{t}}}\ge 0;s}_{io2.1}^{t-}\ge 0, { s}_{ro2.1}^{t+}\ge 0$$

$${Z}_{do{\left({2,1}\right)}}^{t}=\sum_{o=1}^{n}{Z}_{do{\left(2, 1\right)}_{ln}}^{t}{\uplambda }_{{\text{do}}2}^{{\text{t}}}-{s}_{do{\left(2, 1\right)}}^{t-}$$(d = 1…D)

$${\text{e}}{\lambda }_{0k}^{t}=1 (i=1,\dots ,{m}_{k})$$

$${Z}_{co{k}_{l}}^{\left(t,t+1\right)}=\sum_{j=1}^{n}{Z}_{co{k}_{l}}^{\left(t,t+1\right)}{\uplambda }_{c{ok}_{l}}^{{\text{t}}}+{s}_{c{ok}_{l}}^{t\left(t,t+1\right)}$$(c = 1…C)

$${s}_{c{ok}_{l}}^{t\left(t,t+1\right)}\ge 0$$

Period and division efficiencies:

The period and division efficiencies are as follows.

Period efficiency:

$${\partial }_{0}^{*}={\text{min}}\frac{{\sum }_{k=1}^{K}{W}^{k}\left[1-\frac{1}{{m}_{k}+{ninput}_{k}}({\sum }_{i=1}^{{m}_{k}}\frac{{S}_{iok}^{t-}}{{x}_{iok}^{t}}+{\sum }_{{k}_{l}}^{{ninput}_{k}}\frac{{s}_{o{k}_{l}}^{(t,t+1)}}{{z}_{o{k}_{l}}^{(t,t+1)}})\right]}{{\sum }_{k=1}^{K}{W}^{k}\left[1+\frac{1}{{r}_{1k}+{r}_{2k}+{link}_{k}}(\sum_{r=1}^{{r}_{1k}}\frac{{s}_{rokgood}^{t+}}{{y}_{rokgood}^{t}}+\sum_{r=1}^{{r}_{2k}}\frac{{s}_{rokbad}^{t-}}{{y}_{rokbad}^{t}}+ {\sum }_{(kl)}^{link}\frac{{s}_{o(kl)}^{t}}{{Z}_{o(kl)}^{t}})\right]}$$

Division efficiency:

$${\varphi }_{0}^{*}={\text{min}}\frac{{\sum }_{t=1}^{T}{W}^{t}\left[1-\frac{1}{{m}_{k}+{ninput}_{k}}({\sum }_{i=1}^{{m}_{k}}\frac{{S}_{iok}^{t-}}{{x}_{iok}^{t}}+{\sum }_{{k}_{l}}^{{ninput}_{k}}\frac{{s}_{o{k}_{l}}^{(t,t+1)}}{{z}_{o{k}_{l}}^{(t,t+1)}})\right]}{{\sum }_{t=1}^{T}{W}^{t}\left[1+\frac{1}{{r}_{1k}+{r}_{2k}+{link}_{k}}(\sum_{r=1}^{{r}_{1k}}\frac{{s}_{rokgood}^{t+}}{{y}_{rokgood}^{t}}+\sum_{r=1}^{{r}_{2k}}\frac{{s}_{rokbad}^{t-}}{{y}_{rokbad}^{t}}+ {\sum }_{(kl)}^{link}\frac{{s}_{o(kl)}^{t}}{{Z}_{o(kl)}^{t}})\right]}$$

Division period efficiency:

$${\rho }_{0}^{*}={\text{min}}\frac{1-\frac{1}{{m}_{k}+{ninput}_{k}}({\sum }_{i=1}^{{m}_{k}}\frac{{S}_{iok}^{t-}}{{x}_{iok}^{t}}+{\sum }_{{k}_{l}}^{{ninput}_{k}}\frac{{s}_{o{k}_{l}}^{(t,t+1)}}{{z}_{o{k}_{l}}^{(t,t+1)}} )}{1+\frac{1}{{r}_{1k}+{r}_{2k}+{link}_{k}}(\sum_{r=1}^{{r}_{1k}}\frac{{s}_{rokgood}^{t+}}{{y}_{rokgood}^{t}}+\sum_{r=1}^{{r}_{2k}}\frac{{s}_{rokbad}^{t-}}{{y}_{rokbad}^{t}}+ {\sum }_{(kl)}^{link}\frac{{s}_{o(kl)}^{t}}{{Z}_{o(kl)}^{t}})}$$

From the above results, we obtain overall efficiency, period efficiency, division efficiency, and division period efficiency.

### Input, desirable output, and undesirable output efficiencies

We use the Hu and Wang [26] and Hu and Chang [27] total-factor energy efficiency index to overcome any possible biases in the traditional efficiency indicators. In the social security stage, the input variables are those insured under government social health insurance and net total social security expenditure, and the output variables are net social security benefits and SDG3.4 preventable and treatable mortality. In the healthcare stage, the input variables are physicians and healthcare expenditure, and the output variables are hospital discharges, deaths (undesirable output), and SDG3.1 maternal mortality (undesirable output). “I” represents area, and “t” represents time. The efficiency models are defined as follows.

$${\text{Input}}\,{\text{efficiency}} = \frac{{{\text{Target}}\,{\text{input}}}}{{{\text{Actual}}\,{\text{input}}}}$$

$${\text{Output}}\,{\text{efficiency}} = \frac{{{\text{Actual}}\,{\text{Desirable}}\,{\text{output}}}}{{{\text{Target}}\,{\text{Desirable}}\,{\text{output}}}}$$

If the target inputs equal the actual inputs, then the efficiencies are 1, which indicates overall efficiency. However, if the target inputs are less than the actual inputs, then the efficiencies are less than 1, which indicates overall inefficiency.

If the target desirable outputs are equal to the actual desirable outputs, then the efficiencies are 1, indicating overall efficiency. However, if the target desirable outputs are more than the actual desirable outputs, then the efficiencies are less than 1, indicating overall inefficiency.

### Empirical analysis

**Fig. 1:**
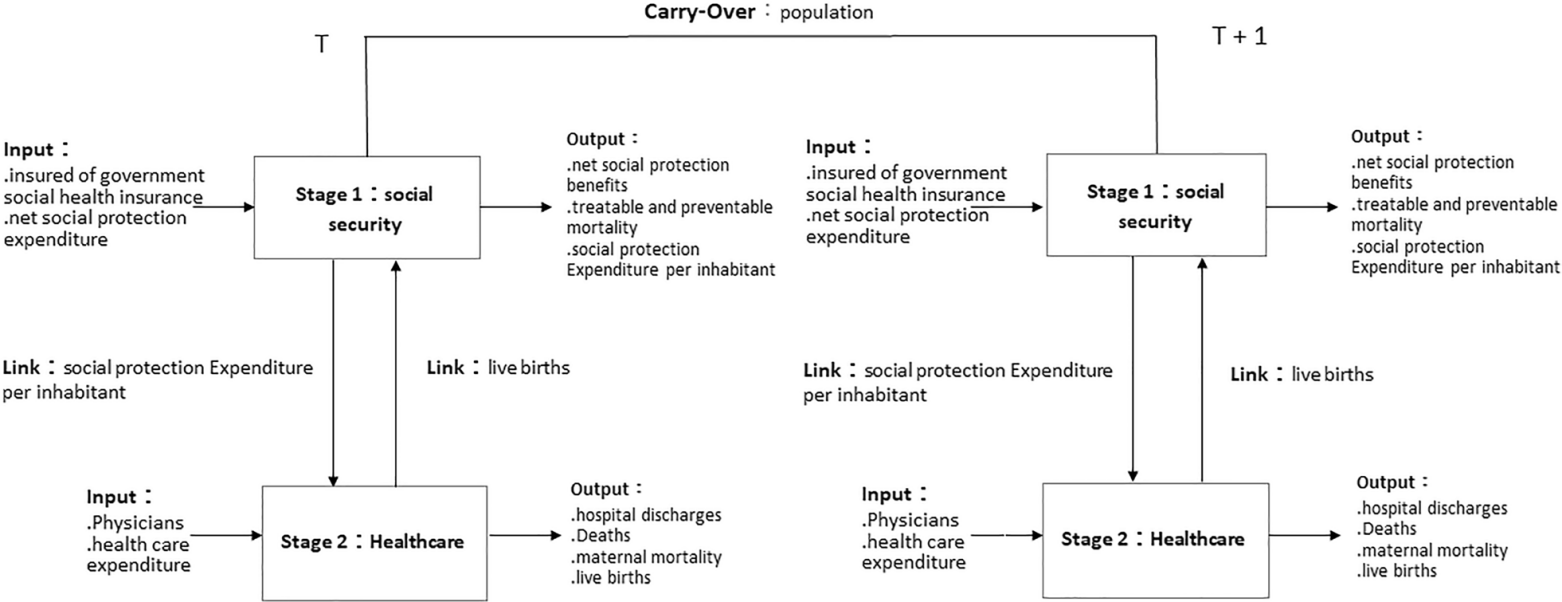
General overview of variables and carry-over

We take 26 countries of OECD as DMUs and use 5-year data from 2013 to 2017 taken from Eurostat and OECD databases. Due to a lack of data, Australia, Canada, Chile, Colombia, Costa Rica, Israel, Japan, Mexico, New Zealand, South Korea, Spain, and the United States are excluded. There are 25 European countries and 1 Asian country by geographical regions. In the social security stage, the number of beneficiaries and net social security expenditure are adopted as the input variables. Net social security payments and preventable and treatable mortality are the output variables. The per capita social security expenditure serves as a link between the social security stage and the healthcare stage. In the healthcare stage, physicians and healthcare expenditure are taken as input variables, while the output variables are hospital discharges, deaths (undesirable output), and SDG3.1 maternal deaths (undesirable output). Live births serve as a link between the healthcare stage and the social security stage, as they are an output in the healthcare stage that cycles back as an input in the social security stage. Population is a dynamic carry-over variable across periods (see Fig. 1 and Table 2).

**Table 2:** Variable description

Variable			Unit	Reason for selection	References
Social security	Input	Insured by government social health insurance	1000 people	Basic labor input of social security; number of insured by government social health insurance	Antonelli and Bonis [4]
		Net social protection expenditure	Million EUR	Basic input of social security, which is the substitution variable for social insurance premiums	Dutu and Sicari [15]
	Output	Net social protection benefits	Million EUR	SDG1.3 is the basic output of social security	Halaskova et al. [21]
		Treatable and preventable mortality	%	SDG3.4 represents public health and primary preventive care outcomes	Rutstein et al. [50], Lagasse et al. [32]
Links		Social protection expenditure per inhabitant	EUR	Link to healthcare stage	
Health-care	Input	Physicians	Persons	Basic labor input of healthcare stage	Afonso and St. Aubyn [2]
		Healthcare expenditure	Million EUR	Basic capital input of healthcare can be observed in the differences in health between countries	Cos and Moral-Benito [13], Samut and Cafri [51], Top et al. [60]
	Output	Maternal mortality (OB)	Per 100,000 mothers	SDG3.1 is the negative impact of healthcare	Mustapha and Sahand [40]
	Undesirable	Hospital discharges	Per 100,000 inhabitants	Basic output of healthcare	Samut and Cafri [51], Ozcan and Khushalani [46]
		Deaths (OB)	Persons	Negative impact of healthcare	Cos and Moral-benito [13]
Link		Live births	Persons	SDG3.2 is the basic output of healthcare, which circulate backs as input to the social security stage	Afonso and St. Aubyn [2], Adams et al. [1]
Carry-over		Population	Persons	Observe the impact of population changes on social security and healthcare efficiency	Chiu et al. [9]

Table 3 shows that compared to NHS, NHI has higher preventable and treatable mortality, along with lower per capita social security expenditure, healthcare expenditure, and maternal deaths. Both preventable and treatable mortality and maternal deaths exhibit higher variances in NHS, indicating a greater variation in sustainable outcomes related to ensuring health and well-being compared to NHI. Additionally, NHS demonstrates higher variances in all variables in the healthcare stage, suggesting greater disparities in healthcare outcomes compared to NHI from a healthcare perspective. Table 4 shows that countries with per capita GDP over US$41,500 have higher per capita social protection expenditure, healthcare expenditure, and hospital discharges, along with lower preventable and treatable mortality and maternal deaths compared to per capita GDP below US$41,500. Both preventable and treatable mortality and maternal deaths exhibit higher variances in countries with per capita GDP below US$41,500, implying significant variations in sustainable outcomes related to ensuring health and well-being from a sustainable business perspective. From the perspective of per capita social protection expenditure and healthcare expenditure, countries with per capita GDP over US$41,500 demonstrate greater differences in per capita social protection expenditure and healthcare expenditure compared to countries with per capita GDP below US$41,500. Interestingly, countries with per capita GDP over US$41,500 exhibit higher variances in the undesirable outputs of deaths and hospital discharges compared to countries with per capita GDP below US$41,500. This suggests that countries with per capita GDP exceeding US$41,500 show greater differences in the outcomes of deaths and hospital discharges, compared to countries with per capita GDP below US$41,500.

**Table 3:** Descriptive statistics for the classification of healthcare systems

Independent variable			Mean		Standard deviation		Minimum		Maximum	
			NHI	NHS	NHI	NHS	NHI	NHS	NHI	NHS
Social security	Input	Insured by government social health insurance (1000 people)	148,762.11	23,758.95	494,829.28	29,314.17	324.00	602.00	2,023,825.00	80,189.10
		Net social protection expenditure (million EUR)	139,556.12	158,257.26	213,154.36	240,929.49	2,434.37	5,311.53	749,292.40	879,550.55
	Output	Net social protection benefits (million EUR)	133,381.51	151,330.56	203,507.03	229,647.35	2,409.32	4,991.60	695,663.25	839,259.82
		Treatable and preventable mortality (%)	284.24	87.55	106.54	121.49	161.38	175.07	581.64	579.86
Links		Social protection expenditure per inhabitant (eur)	8350.59	9185.65	5564.75	6224.23	1660.53	1102.15	20,550.69	20,844.65
Healthcare	Input	Physicians (persons)	55,464.35	80,504.96	58,848.15	107,707.08	1168.00	1537.00	351,195.00	351,195.00
		Healthcare expenditure (million EUR)	52,736.31	68,157.55	77,824.86	97,701.23	991.93	2146.52	369,091.00	369,091.00
	Output	Maternal mortality (per 100,000 maternal)	6.90	7.33	6.96	7.01	1.30	1.20	55.20	32.40
	Undesirable	Hospital discharges (per 100,000 inhabitant)	15,797.54	17,051.98	3,017.27	4,437.94	8,453.10	9,247.10	20,627.40	25,685.90
		Deaths (persons)	159,735.35	217,590.76	189,932.73	288,096.46	2,049.00	3,822.00	606,410.00	932,272.00
Link		Live births (persons)	148,762.11	276,292.47	250,938.52	398,589.42	4,034.00	6,050.00	819,328.00	1,337,504.00
Carry-over		Population (persons)	139,556.12	24,297,549.47	21,012,634.55	30,904,754.48	321,857.00	537,039.00	66,809,816.00	82,521,653.00

Source: Eurostat and OECD databases

**Table 4:** Descriptive statistics for the classification of per capita GDP

Independent variable			Mean		Standard deviation		Minimum		Maximum	
			GDP > 4.15	GDP < 4.15	GDP > 4.15	GDP < 4.15	GDP > 4.15	GDP < 4.15	GDP > 4.15	GDP < 4.15
Social security	Input	Insured by government social health insurance (1000 people)	18,755.11	172,997.21	23,349.41	528,167.21	602.00	324.00	73,891.00	2,023,825.00
		Net social protection expenditure (million EUR)	163,987.31	130,948.98	262,188.72	179,964.76	5,311.53	2,434.37	879,550.55	697,572.79
	Output	Net social protection benefits (million EUR)	154,769.32	127,181.36	246,949.08	176,559.38	4,991.60	2,409.32	839,259.82	691,876.42
		Treatable and preventable mortality (%)	266.08	305.21	96.72	124.30	161.38	175.07	579.86	581.64
Links		Social protection expenditure per inhabitant (eur)	9294.00	8113.77	6422.70	5184.60	1820.52	1102.15	20,844.65	16,308.24
Healthcare	Input	Physicians (persons)	70,281.28	61,835.63	93,678.07	72,742.55	1,537.00	1,168.00	351,195.00	241,512.00
		Healthcare expenditure (million EUR)	64,246.88	54,274.48	102,415.39	68,085.26	2,146.52	991.93	369,091.00	261,567.48
	Output	Maternal mortality (per 100,000 mothers)	6.49	7.68	5.93	7.85	1.50	1.20	32.40	55.20
	Undesirable	Hospital discharges (per 100,000 inhabitants)	17,199.68	15,456.84	4118.96	3068.59	8453.10	9247.10	25,685.90	20,396.10
		Deaths (persons)	194,244.51	174,180.77	262,720.90	210,178.05	3,822.00	2,049.00	932,272.00	649,061.00
Link		Live births (persons)	199,732.37	249,364.32	261,271.05	375,943.59	6,050.00	4,034.00	819,328.00	1,337,504.00
Carry-over		Population (person)	19,418,159.29	20,646,260.45	25,262,146.09	26,543,306.50	537,039.00	321,857.00	82,521,653.00	79,814,871.00

Source: Eurostat and OECD databases

Table 5 shows that the overall average efficiency is 0.662. The average efficiencies for the years 2013 to 2017 are 0.757, 0.683, 0.704, 0.727, and 0.694, respectively, with a slight difference of approximately 0.074. The improvement space ranges from 0.243 to 0.317. The countries with consistently high efficiency values of 1 over the 5-year period are Estonia, Iceland, Latvia, Luxembourg, Poland, and Turkey. The countries with lower performance are France (0.225), Greece (0.220) and Portugal (0.172). These countries share the common characteristic of having an aging population structure, with the proportion of people aged 65 and above higher than the OECD average. In particular, the efficiency value of Italy was 1 in 2013 and 2014, but only 0.339 in 2017. The efficiency value of Austria was 1 in 2013, only 0.411 in 2014, and returned to 0.605 in 2017. The efficiency value of Germany was 0.599, dropped to 0.510 and improved to 1 in 2017.

**Table 5:** Efficiency values

DMU	2013	2014	2015	2016	2017	Efficiency value	Ranking
Austria	1.000	0.411	0.408	0.452	0.605	0.522	17
Belgium	0.565	0.456	0.466	0.516	0.513	0.400	20
Czech Republic	0.843	0.410	0.363	0.396	0.405	0.337	22
Denmark	0.677	0.617	0.700	0.840	0.814	0.705	12
Estonia	1.000	1.000	1.000	1.000	1.000	1.000	1
Finland	0.763	0.520	0.571	0.496	0.377	0.518	18
France	0.287	0.386	0.326	0.401	0.261	0.225	24
Germany	0.599	0.510	1.000	1.000	1.000	0.745	11
Greece	0.222	0.379	0.272	0.322	0.288	0.220	25
Hungary	0.730	0.532	0.818	0.751	0.749	0.682	13
Iceland	1.000	1.000	1.000	1.000	1.000	1.000	1
Ireland	0.462	0.730	0.708	0.374	0.736	0.530	16
Italy	1.000	1.000	0.382	0.687	0.339	0.418	19
Latvia	1.000	1.000	1.000	1.000	1.000	1.000	1
Lithuania	1.000	1.000	1.000	1.000	0.873	0.974	7
Luxembourg	1.000	1.000	1.000	1.000	1.000	1.000	1
Netherlands	0.406	0.300	0.275	0.378	0.691	0.326	23
Norway	1.000	1.000	1.000	1.000	0.833	0.965	8
Poland	1.000	1.000	1.000	1.000	1.000	1.000	1
Portugal	0.184	0.211	0.178	0.211	0.194	0.172	26
Slovak Republic	0.770	0.736	0.775	0.582	0.779	0.682	14
Slovenia	0.835	0.820	1.000	1.000	1.000	0.924	9
Sweden	0.331	0.461	0.425	0.720	0.378	0.388	21
Switzerland	1.000	0.596	0.635	0.772	0.534	0.661	15
Turkey	1.000	1.000	1.000	1.000	1.000	1.000	1
United Kingdom	1.000	0.675	1.000	1.000	0.676	0.815	10
Efficiency value	0.757	0.683	0.704	0.727	0.694	0.662	

Table 6 shows that the efficiency value of NHI of 0.746 is higher than that of NHS of 0.668. Except for 2014, the efficiency values of NHI in other years are higher than those of NHS. The reason NHI’s efficiency value was lower than NHS in 2014 is due to the greater decrease in efficiency across the two variables, treatable and preventable mortality and physicians in NHI. In NHI the efficiency values for treatable and preventable mortality and physicians decreased by 0.200 and 0.107, respectively, while in NHS, the efficiency values for these two variables decreased by only 0.010 and 0.046, respectively. The efficiency value of countries with per capita GDP greater than US$41,500 is 0.760 higher than that of countries with per capita GDP less than US$41,500. Interestingly, the same variance is for per capita GDP of poorer countries less than US$41,500 and per capita GDP of richer countries higher than US$41,500. Afonso et al. [3] noted under different norms that countries with relatively expensive resources may be wrongly regarded as inefficient. Therefore, this study shows that the possible reason for the low efficiency of countries with per capita GDP greater than US$41,500 is that various inputs are relatively expensive, which leads to relatively low efficiency.

**Table 6:** Efficiency values for each year classified by health system and per capita GDP

Efficiency values					
	2013	2014	2015	2016	2017
NHI	0.802	0.677	0.738	0.750	0.761
NHS	0.694	0.690	0.658	0.695	0.603
GDP > 4.15	0.699	0.614	0.655	0.688	0.672
GDP < 4.15	0.814	0.751	0.753	0.765	0.716

Descriptive statistics				
	NHI	NHS	GDP > 4.15	GDP < 4.15
Mean	0.746	0.668	0.666	0.760
Standard deviation	0.045	0.040	0.033	0.036
Variance	0.002	0.002	0.001	0.001
Minimum	0.677	0.603	0.614	0.716
Maximum	0.802	0.695	0.699	0.814

Table 7 shows that the efficiency value of the healthcare stage at 0.752 is slightly higher than the efficiency value of the social security stage at 0.674, indicating that the healthcare stage has slightly better efficiency comparatively. There is a significant difference in efficiency between countries in the social security and healthcare stages, which confirms the findings of Dutu and Sicari [15] regarding the large variations in efficiency of public welfare expenditures in the healthcare and public service sectors among OECD countries.

**Table 7:** Efficiency in social security and healthcare stages across countries

DMU	2013		2014		2015		2016		2017		Efficiency value			
	I	II	I	II	I	II	I	II	I	II	I	Ranking	II	Ranking
Austria	1.000	1.000	0.653	0.170	0.435	0.381	0.450	0.455	0.379	0.831	0.583	16	0.567	19
Belgium	0.391	0.740	0.240	0.671	0.304	0.629	0.308	0.724	0.246	0.779	0.298	22	0.709	16
Czech Republic	0.686	1.000	0.246	0.574	0.235	0.491	0.196	0.596	0.194	0.615	0.311	21	0.655	18
Denmark	0.596	0.759	0.567	0.667	0.629	0.772	0.681	1.000	0.629	1.000	0.620	14	0.840	11
Estonia	1.000	1.000	1.000	1.000	1.000	1.000	1.000	1.000	1.000	1.000	1.000	1	1.000	1
Finland	0.526	1.000	0.420	0.621	0.538	0.603	0.534	0.457	0.409	0.346	0.485	18	0.605	19
France	0.178	0.396	0.232	0.540	0.120	0.531	0.189	0.613	0.128	0.394	0.169	25	0.495	24
Germany	0.906	0.293	0.368	0.653	1.000	1.000	1.000	1.000	1.000	1.000	0.855	12	0.789	12
Greece	0.154	0.289	0.219	0.539	0.184	0.359	0.156	0.487	0.150	0.426	0.173	24	0.420	25
Hungary	1.000	0.459	0.468	0.596	0.893	0.743	0.723	0.779	0.948	0.550	0.806	13	0.625	17
Iceland	1.000	1.000	1.000	1.000	1.000	1.000	1.000	1.000	1.000	1.000	1.000	1	1.000	1
Ireland	0.491	0.434	0.459	1.000	0.416	1.000	0.349	0.400	0.472	1.000	0.437	19	0.767	13
Italy	1.000	1.000	1.000	1.000	0.180	0.583	0.719	0.655	0.128	0.550	0.605	15	0.758	14
Latvia	1.000	1.000	1.000	1.000	1.000	1.000	1.000	1.000	1.000	1.000	1.000	1	1.000	1
Lithuania	1.000	1.000	1.000	1.000	1.000	1.000	1.000	1.000	0.991	0.756	0.998	10	0.951	8
Luxembourg	1.000	1.000	1.000	1.000	1.000	1.000	1.000	1.000	1.000	1.000	1.000	1	1.000	1
Netherlands	0.295	0.517	0.238	0.361	0.194	0.356	0.257	0.499	0.383	1.000	0.273	23	0.547	22
Norway	1.000	1.000	1.000	1.000	1.000	1.000	1.000	1.000	1.000	0.666	1.000	1	0.933	9
Poland	1.000	1.000	1.000	1.000	1.000	1.000	1.000	1.000	1.000	1.000	1.000	1	1.000	1
Portugal	0.155	0.213	0.176	0.245	0.152	0.203	0.143	0.278	0.132	0.256	0.152	26	0.239	26
Slovak Republic	0.539	1.000	0.472	1.000	0.550	1.000	0.372	0.791	\	1.000	0.498	17	0.958	7
Slovenia	1.000	0.669	1.000	0.640	1.000	1.000	1.000	1.000	1.000	1.000	1.000	1	0.862	10
Sweden	0.289	0.374	0.264	0.658	0.369	0.482	0.813	0.626	0.246	0.509	0.396	20	0.530	23
Switzerland	1.000	1.000	0.694	0.499	1.000	0.269	1.000	0.544	0.589	0.479	0.857	11	0.558	21
Turkey	1.000	1.000	1.000	1.000	1.000	1.000	1.000	1.000	1.000	1.000	1.000	1	1.000	1
United Kingdom	1.000	1.000	1.000	0.349	1.000	1.000	1.000	1.000	1.000	0.352	1.000	1	0.740	15
Efficiency value	0.739	0.775	0.643	0.722	0.662	0.746	0.688	0.766	0.638	0.750	0.674		0.752	

The top-ranked countries in the social security stage with efficiency values of 1 for each year include Estonia, Iceland, Latvia, Luxembourg, Norway, Poland, Slovenia, Turkey, and the United Kingdom. Among them, Iceland and Poland have the same performance as reported by Hsu et al. [24]. The countries ranking lower in efficiency are Greece (0.173), France (0.169), Portugal (0.152), and the Netherlands (0.273). The common reasons for their low efficiency are poor outcomes in preventable and treatable mortality and per capita social security expenditure. The low efficiency of France and the Netherlands aligns with the findings of Adams et al. [1]. The top-ranked countries in the healthcare stage with efficiency values of 1 for each year are Estonia, Iceland, Latvia, Luxembourg, Poland, and Turkey. The high efficiency of Estonia, Iceland, and Turkey aligns with the findings of Lu et al. [36]. The high efficiency of Turkey and Poland is consistent with Samut and Cafri [51]. The countries ranking lower in efficiency in the healthcare stage include Portugal (0.239), Greece $(0.420)$, and France (0.495). The lower efficiency of France corresponds to the findings of Adams et al. [1] and Samut and Cafri [51]. The common reasons for the lower efficiency values in Greece, France, and Portugal are underperforming outcomes in discharges and healthcare expenditure.

When assessing dynamic overall efficiency using population as a carry-over, we see that countries with good efficiency in the social security stage may not necessarily have the same level of efficiency in the healthcare stage. For example, the United Kingdom, Slovenia, and Switzerland show this trend. The United Kingdom is the most prominent example, ranking first in social security efficiency with a value of 1 over the five-year period, but ranking ${15}^{0.74}$ in the healthcare stage. On the other hand, countries with lower efficiency in the social security stage may perform well in the healthcare stage. For instance, the Slovak Republic ranks 1${7}^{0.498}$ in social security efficiency over the five-year period, but ranks ${7}^{0.958}$ in the healthcare stage.

Table 8 shows in the social security stage that NHI has a slightly higher efficiency value of 0.710 compared to NHS at 0.624. In the healthcare stage, NHI also exhibits a slightly higher efficiency value of 0.781 compared to NHS at 0.712. This indicates that NHI has slightly higher efficiency in both social security and healthcare compared to NHS. In the social security stage, countries with per capita GDP less than US$41,500 have an efficiency value of 0.734, which is higher than countries with per capita GDP greater than US$41,500, with a value of 0.613. In the healthcare stage, countries with per capita GDP less than US$41,500 have an efficiency value of 0.785, or higher than countries with per capita GDP greater than US$41,500 at 0.718. This indicates that countries with lower per capita GDP have higher efficiency in both social security and healthcare compared to countries with higher per capita GDP.

**Table 8:** Descriptive statistics of efficiency values classified by healthcare system and per capita GDP

	Social security		Healthcare		Social security		Healthcare	
	NHI	NHS	NHI	NHS	GDP > 4.15	GDP < 4.15	GDP > 4.15	GDP < 4.15
Mean	0.710	0.624	0.781	0.712	0.613	0.734	0.718	0.785
Standard deviation	0.319	0.331	0.196	0.243	0.299	0.342	0.183	0.246
Variance	0.102	0.110	0.038	0.059	0.089	0.117	0.034	0.060
Minimum	0.169	0.152	0.495	0.239	0.169	0.152	0.495	0.239
Maximum	1.000	1.000	1.000	1.000	1.000	1.000	1.000	1.000

**Fig. 2:**
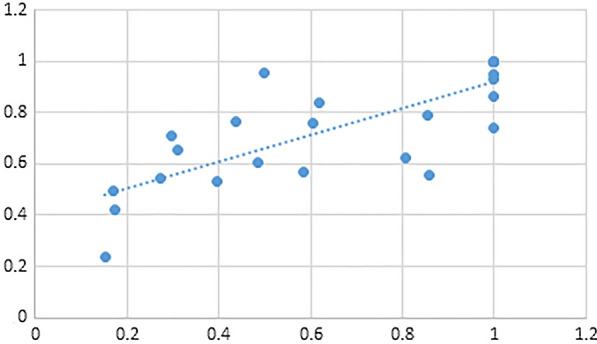
Correlation analysis

In Fig. 2, the efficiency in the social security stage and efficiency in the healthcare stage shows a high positive correlation coefficient of 0.785. This indicates that as efficiency in the social security stage improves, healthcare efficiency also tends to be better. This finding supports the research conducted by González et al. [18], who found a positive correlation between healthcare efficiency and the percentage of government healthcare expenditure out of total healthcare expenditure. It is also in line with the research by Ozcan and Khushalani [46], who suggested that countries with high efficiency in public healthcare tend to have better overall healthcare system efficiency.

Countries with per capita GDP less than US$41,500 have higher social security and healthcare efficiency than countries with per capita GDP greater than US$41,500. The possible reason is that the efficiency values of countries with per capita GDP less than US$41,500 are better in preventable and treatable mortality and healthcare expenditure, and the number of hospital discharges is higher than countries with per capita GDP greater than US$41,500. Countries with per capita GDP less than US$41,500 have higher social security efficiency values than countries with per capita GDP greater than US$41,500, indicating that low-income countries with fewer resources may not necessarily have poorer efficiency values. One possible reason why NHI social security and healthcare efficiency values are higher than NHS is that NHI efficiency is greater than NHS in terms of preventable and treatable mortality and discharge numbers.

**Fig. 3:**
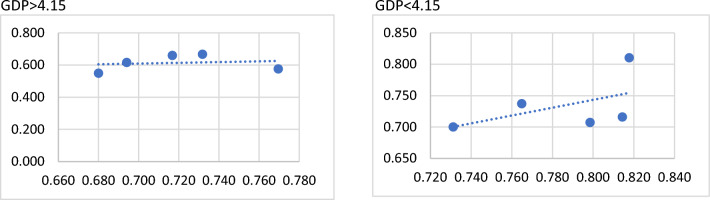
Correlation analysis between GDP > 4.15 and GDP < 4.15 for social security stage and healthcare stage

**Fig. 4:**
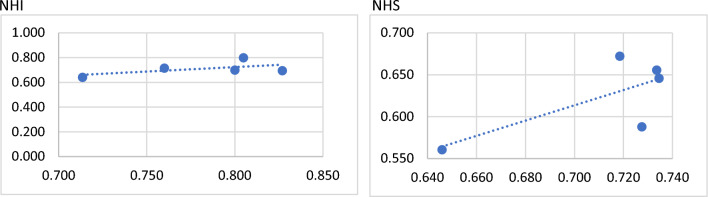
Correlation analysis of social security stage and healthcare stage of NHI and NHS

For correlation analysis, a correlation coefficient of 0.513 for Countries with per capita GDP less than US$41,500 is a moderate positive correlation. This means that countries with per capita GDP less than US$41,500 have higher social security efficiency and better healthcare efficiency. A correlation coefficient of 0.561 for NHI is moderately positive, and a correlation coefficient of 0.715 for NHS is highly positive. It means that the higher the social security efficiency of NHI and NHS is, the better is healthcare efficiency. (See Figs. 3 and 4).

In Table 9, the NHI efficiency value of 0.669 for preventable and treatable mortality is higher than the NHS efficiency value of 0.552. The efficiency value of 0.526 for countries with per capita GDP greater than US$41,500 is lower than 0.712 for countries with per capita GDP less than US$41,500. This implies that countries with rich resources and high national income may not have better efficiency in SDG3.4 preventable and treatable mortality than low-income countries with fewer resources. Ruhm [48, 49] found that a better economic situation may lead to poorer health, and Mackenbach [37] stated that countries with high per capita healthcare expenditures do not show a relatively high level of mortality reduction. However, unlike Treuniet et al. [61] who found that areas with more healthcare care resources have lower mortality rates from avoidable diseases, this part of the research is still inconclusive. Our study believes that one possible reason for the higher efficiency of preventable and treatable mortality in countries with per capita GDP of less than US$41,500 is the higher healthcare efficiency.

**Table 9:** Analysis of health system and per capita GDP of preventable and treatable mortality

Efficiency values					
	2013	2014	2015	2016	2017
NHI	0.778	0.578	0.682	0.652	0.654
NHS	0.588	0.578	0.510	0.591	0.490
GDP > 4.15	0.588	0.447	0.535	0.578	0.484
GDP < 4.15	0.807	0.709	0.684	0.675	0.685

Descriptive statistics				
	NHI	NHS	GDP > 4.15	GDP < 4.15
Mean	0.669	0.552	0.526	0.712
Standard deviation	0.072	0.048	0.060	0.055
Variance	0.005	0.002	0.004	0.003
Minimum	0.578	0.490	0.447	0.675
Maximum	0.778	0.591	0.588	0.807

## Conclusion

This paper allows OECD countries to evaluate the efficiency of social security and healthcare from the perspective of sustainable development and to explore whether the efficiency of social security operation affects healthcare efficiency. As the global population ages, the average health expenditures among OECD countries increased from 7.8% of GDP in 2005 to 9.9% in 2020, and social security and welfare expenditures rose from 14.5% of GDP in 1980 to 20.2% in 2016. However, social security and healthcare relate to SDG1, SDG3 and SDG10. This research uses two-stage recycle dynamic undesirable SBM DEA to observe the efficiency of social security and healthcare in OECD countries. The main empirical results, findings, and recommendations are as follows.

1. The five-year overall efficiency value is 0.662, and the efficiency difference between each year is 0.074. There is still room for improvement from 0.243 to 0.317. The best-performing countries with an efficiency value of 1 are Estonia, Iceland, Latvia, Luxembourg, Poland, and Turkey. What the worst three countries France (0.225), Greece (0.220), and Portugal (0.172) have in common is that their efficiency values of preventable and treatable mortality, per capita social security expenditure, and number of hospital discharges lag behind. This means that countries with abundant resources and high national income may not necessarily have better healthcare outcomes, while countries with less resources and low income may not necessarily suffer poorly. There may be great differences in the cost-effectiveness of healthcare service systems.
2. The efficiency value of 0.674 in the social security stage is slightly lower than that of 0.752 in the healthcare stage, and countries with good efficiency in the former stage may not be as good in the latter stage, such as United Kingdom, Slovenia, and Switzerland. We find that the higher is social security efficiency, the better is healthcare efficiency of countries with GDP per capita below US$41,500. The efficiency values of countries with per capita GDP less than US$41,500 are higher than those with per capita GDP greater than US$41,500, which may be due to the fact that the efficiency values of countries with per capita GDP less than US$41,500 in preventable and treatable mortality, healthcare care expenditure, and hospital discharge are higher than those for countries with per capita GDP greater than US$41,500. The efficiency of NHI in both stages is higher than that of NHS, which may be caused by the fact that the efficiency of NHI in preventable and treatable mortality and discharge number is higher than that of NHS.
3. The higher the social security efficiency is for NHS countries, the better is healthcare efficiency. When the financial source of the social security system is taxation, it is more likely to bring higher efficiency to healthcare.
4. The efficiency values of SDG3.4 preventable and treatable mortality and healthcare stage of the countries with GDP per capita below US$41,500 are higher than those with GDP per capita over US$41,500. Clearly, high-income countries with abundant resources may not necessarily have better healthcare efficiency.

### Recommendations

With the rising ratio of health care expenditure to GDP in OECD countries, identifying mechanisms to improve healthcare efficiency or exploring the reasons for inefficiency has become an important challenge faced by various OECD countries. As such, this study puts forward the following recommendations.

1. Countries with per capita GDP below US$41,500 and with a NHI system can improve healthcare efficiency by enhancing social security efficiency. The study finds a strong positive correlation between social security efficiency and healthcare efficiency. The higher the social security efficiency is in countries with per capita GDP below US$41,500, the better is healthcare efficiency. NHI countries and NHS countries with higher social security efficiency also exhibit better healthcare efficiency.
2. Countries with per capita GDP over US$41,500 should be more proactive in addressing SDG3.4 preventable and treatable mortality. The performances of healthcare and preventable and treatable mortality in countries with per capita GDP over US$41,500 are not better versus countries with per capita GDP below US$41,500. It is important to carefully examine healthcare expenditure to improve efficiency.
3. This study only considers the impact of social security on healthcare efficiency. However, private insurance also plays an important role in healthcare systems. Countries with per capita GDP over US$41,500 are expected to have more developed private insurance systems. It is recommended that future research consider incorporating variables related to private insurance to assess the efficiency landscape of high and low GDP countries.

## Data Availability

Not applicable.

## Appendix

Table: Classification of the health care systems of 26 OECD countries

**Table:**

NHS 11	NHI 15
Denmark Finland Greece Iceland Ireland Italy Latvia Norway Portugal Sweden United Kingdom	Austria Belgium Czech Republic Estonia France Germany Hungry Lithuania Luxembourg Netherlands Poland Slovak Republic Slovenia Switzerland Turkey

Countries in different system by Böhm et al. [6] and Viera Ivanková et al. [28]
